# Effects of lifestyle and single nucleotide polymorphisms on breast cancer risk: a case–control study in Japanese women

**DOI:** 10.1186/1471-2407-13-565

**Published:** 2013-12-01

**Authors:** Taeko Mizoo, Naruto Taira, Keiko Nishiyama, Tomohiro Nogami, Takayuki Iwamoto, Takayuki Motoki, Tadahiko Shien, Junji Matsuoka, Hiroyoshi Doihara, Setsuko Ishihara, Hiroshi Kawai, Kensuke Kawasaki, Youichi Ishibe, Yutaka Ogasawara, Yoshifumi Komoike, Shinichiro Miyoshi

**Affiliations:** 1 Department of General Thoracic Surgery and Breast and Endocrinological Surgery, Okayama University Graduate School of Medicine, Dentistry, and Pharmaceutical Sciences, 2-5-1 Shikata-cho, Okayama-city, Okayama 700-8558, Japan; 2 Department of Radiology, Okayama Saiseikai General Hospital, 1-17-18 Ifuku-cho, Okayama-city, Okayama 700-8511, Japan; 3 Department of Breast Surgery, Okayama Rousai Hospital, 1-17-18 Chikkoumidorimachi, Okayama-city, Okayama 702-8055, Japan; 4 Department of Breast Surgery, Kagawa Prefectural Cancer Detection Center, 587-1 Tougou-cho, Takamatu-city, Kagawa 761-8031, Japan; 5 Department of Breast Surgery, Mizushima Kyodo Hospital, 1-1 Mizushima, Minamikasuga-cho Kurashiki-city, Okayama 712-8567, Japan; 6 Department of Breast and Endocrinological Surgery, 5-4-16, Ban-cho, Takamatu-city, Kagawa 760-8557, Japan; 7 Faculty of Medicine, Kinki University Hospital, 377-2 Ohnohigashi, Osakahayama-city, Osaka 589-8511, Japan

**Keywords:** Japanese women, Asian, Breast cancer, Lifestyle, Leisure-time exercise, Parity, Single nucleotide polymorphisms, rs2046210, rs3757318, ESR1

## Abstract

**Background:**

Lifestyle factors, including food and nutrition, physical activity, body composition and reproductive factors, and single nucleotide polymorphisms (SNPs) are associated with breast cancer risk, but few studies of these factors have been performed in the Japanese population. Thus, the goals of this study were to validate the association between reported SNPs and breast cancer risk in the Japanese population and to evaluate the effects of SNP genotypes and lifestyle factors on breast cancer risk.

**Methods:**

A case–control study in 472 patients and 464 controls was conducted from December 2010 to November 2011. Lifestyle was examined using a self-administered questionnaire. We analyzed 16 breast cancer-associated SNPs based on previous GWAS or candidate-gene association studies. Age or multivariate-adjusted odds ratios (OR) and 95% confidence intervals (95% CI) were estimated from logistic regression analyses.

**Results:**

High BMI and current or former smoking were significantly associated with an increased breast cancer risk, while intake of meat, mushrooms, yellow and green vegetables, coffee, and green tea, current leisure-time exercise, and education were significantly associated with a decreased risk. Three SNPs were significantly associated with a breast cancer risk in multivariate analysis: rs2046210 (per allele OR = 1.37 [95% CI: 1.11-1.70]), rs3757318 (OR = 1.33[1.05-1.69]), and rs3803662 (OR = 1.28 [1.07-1.55]). In 2046210 risk allele carriers, leisure-time exercise was associated with a significantly decreased risk for breast cancer, whereas current smoking and high BMI were associated with a significantly decreased risk in non-risk allele carriers.

**Conclusion:**

In Japanese women, rs2046210 and 3757318 located near the ESR1 gene are associated with a risk of breast cancer, as in other Asian women. However, our findings suggest that exercise can decrease this risk in allele carriers.

## Background

Data in the National Statistics of Cancer Registries by Region (1975–2004) indicate that the prevalence of breast cancer in Japan has increased steadily since 1975. More than 60,000 patients had breast cancer in 2008 and the mammary gland is the most common site of a malignant tumor in Japanese women [1]. Additionally, the Vital Statistics Japan database of the Ministry of Health, Labor and Welfare indicates that mortality due to breast cancer in Japan has increased since 1960, with more than 10,000 deaths from breast cancer in 2011 [2].

The relationship of lifestyle factors, including food and nutrition, physical activity, body composition, environmental factors, and reproductive factors, with breast cancer risk have been widely studied, mainly in Europe and the United States, and much evidence linking cancer to these factors has been accumulated. According to the 2007 World Cancer Research Fund/American Institute for Cancer Research (WCRF/AICR) Second Expert Report, the evidence that breastfeeding decreases the breast cancer risk and that alcohol increases this risk is described as “convincing” [3]. In postmenopausal women, evidence that body fat and adult attained height increase breast cancer risk is also stated to be “convincing”. However, the evidence of a relationship of other foods with breast cancer risk remains at the level of “limited-no conclusion”. Thus, it is important to identify risk factors for breast cancer with the goal of prevention through efficient screening and surveillance.

In the United States, a breast cancer risk assessment tool based on a statistical model known as the “Gail model” has been produced by the National Cancer Institute (NCI) [4,5]. However, this model has been developed from epidemiological data in Caucasians and it may be inappropriate to apply the Gail model in the Japanese population [6]. However, there are few epidemiological studies of breast cancer risk in Japanese women and a breast cancer risk model applicable to Japanese women has yet to be established.

Regarding genetic factors, genome-wide association studies (GWAS) have identified several breast cancer susceptibility single nucleotide polymorphisms (SNPs) [7]. However, most of these studies were also conducted in subjects with European ancestry, with some in populations with Chinese ancestry or in African Americans. There is only one such study in subjects with Japanese ancestry. However, allele frequencies related to breast cancer risk and the extent of linkage disequilibrium differ among races. Thus, the validity of the reported associations of SNPs with breast cancer needs to be tested in a Japanese population.

Current findings suggest that the interactions between breast cancer susceptibility SNPs and breast cancer risk are not as strong as those for BRCA1 or BRCA2 gene mutation. However, carriers of risk SNP alleles are more common compared with carriers of BRCA1 or BRCA2 mutation. Evaluation of the need to incorporate SNPs into a breast cancer risk model requires examination of the influence of these SNPs and established breast cancer risk factors to determine whether these are mutually confounding factors. Moreover, such findings might allow risk allele carriers to reduce their incidence of breast cancer through guidance on lifestyle habits.

The current study was performed to add to the relatively small number of studies that have examined genomic factors such as SNPs in combination with non-genomic factors such as those associated with lifestyle. We first aimed to validate whether reported breast cancer susceptibility SNPs are applicable in the Japanese population. We then examined the possible confounding effects on breast cancer risk of SNPs and lifestyle factors such as food, nutrition, physical activity, body composition, environment factors and reproductive factors.

## Methods

### Subjects

A multicenter population-based case–control study was conducted between December 2010 and November 2011 in Japan. The subjects were consecutive patients with non-invasive or invasive breast cancer aged over 20 years old who were treated at Okayama University Hospital, Okayama Rousai Hospital and Mizushima Kyodo Hospital in Okayama and at Kagawa Prefecture Central Hospital in Kagawa. The controls were women aged over 20 years old without a history of breast cancer who underwent breast cancer screening at Mizushima Kyodo Hospital and Okayama Saiseikai Hospital in Okayama and at Kagawa Prefectural Cancer Detection Center in Kagawa. All subjects gave written informed consent before enrollment in the study. A blood sample (5 ml) used for SNP analysis was collected from each subject. Subjects were also given questionnaires that they completed at home and mailed back to Okayama University Hospital. The study was approved by the institutional ethics committee on human research at Okayama University.

### Survey of lifestyle

A survey of lifestyle was performed using an 11-page self-administered questionnaire that included questions on age, height and body weight (current and at 18 years old), cigarette smoking, alcohol drinking, intake of 15 foods items, intake of 4 beverages, leisure-time exercise (current and at 18 years old), menstruation status, age at first menstruation, age at first birth, parity, breastfeeding, age at menopause, hormone replacement therapy (HRT), history of benign breast disease, familial history of breast cancer, and education. Controls answered the survey based on their current status and patients referred to their prediagnostic lifestyle.

Body mass index (BMI) was calculated as body weight/square of height. Former or current alcohol drinkers were asked to give the frequency per week and type of drink usually consumed (beer, wine, sake, whisky, shochu, or others). The alcoholic content of each drink was taken to be 8.8 g per glass (200 ml) of beer, and 20 g per glass of sake (180 ml), wine (180 ml), shochu (110 ml) and whisky (60 ml) [8]. Alcohol intake per day (g/day) was calculated as follows: (total alcohol content per occasion × frequency of consumption per week)/7. Women who currently engaged in leisure-time exercise were asked to give the intensity of physical activity per occurrence and frequency per week. Metabolic equivalent (MET) values of 10, 7, 4, and 3 METs were assigned for strenuous-, moderate-, low-, and very low intensity activities per occurrence, respectively [9], to allow calculation of the intensity of physical activity in leisure-time exercise per week (METs/week). A family history of breast cancer included mother, sisters and daughters (first-degree family history). History of benign breast disease included the non-cancerous breast. Clinical data on patients were obtained from hospital medical records.

### Selection of SNPs

Sixteen breast cancer-associated SNPs were identified from previous GWAS [7] and candidate-gene association studies: ATM/11q22-rs1800054 [10], 8q24-rs1562430 [11], MAP3K1/Chr5-rs889132 [10,12], 2q-rs4666451 [10], 8q24-rs13281615 [10,12,13], TTNT3/11p15-rs909116 [11], 5q-rs30099 [10], IGF1/12q23.2-795399 [10,14], ESR1/6q25.1-rs2046210 [15,16], CAPSP8/2q33-34-rs1045485 [10], 2q35-rs13387042 [10], ESR1/6q25.1-rs3757318 [11], TNRC9/16q12-rs3803662 [12,17], FGFR2/10q26-rs2981282 [10,12], LSP1/11p15.5-rs381798 [12], and HCN1/5p12- rs98178 [10]. Risk alleles associated with breast cancer were identified with reference to the Japanese Single Nucleotide Polymorphism (JSNP) database [18].

### SNP genotyping

Genomic DNA was isolated from whole blood with a Taq-Man® Sample-to-SNP™ kit (Applied Biosystems, Foster City, CA, USA). Samples were analyzed by a TaqMan genotyping assay using the StepOne™ real-time polymerase chain reaction (PCR) system (Applied Biosystems) in a 96-well array plate that included four blank wells as negative controls. The PCR profile consisted of an initial denaturation step at 95°C for 10 min, 40 cycles of 92°C for 15 sec, and 60°C for 1 min. PCR products were analyzed by StepOne™ Software Ver2.01 (Applied Biosystems). To assess the quality of genotyping, we conducted re-genotyping of a randomly selected 5% of samples and obtained 100% agreement.

### Statistical analysis

For all analyses, significance was defined as a p-value <0.05. Associations between lifestyle and breast cancer risk were estimated by computing age adjusted odds ratios (OR) and their 95% confidence intervals (CI) from logistic regression analyses. Height was categorized as ≤150, 151–155, 156–160 and >160 according to quartile. Weight was categorized as <50, 50–54.9, 55–59.9 and ≥60 according to quartile. BMI was categorized as ≤20, 20–21.9, 22–23 and ≥24 according to quartile. Alcohol intake per day (g/day) was categorized as 0, <5, 5–10 and ≥10 g/day according to quartile. Food intake, including meat, fish, egg, soy, milk, fruits, green and yellow vegetables and mushrooms, was categorized as ≤1, 2–4 and 5 times/week. Beverage intake including coffee and green tea was categorized as ≤1, 2–3 and ≥3 cups/day. Intensity of physical activity in leisure time was categorized as 0, <6, 6–11.9, 12–23.9 and ≥24 METs/week. Age at menarche was classified as ≤12, 13 and ≥14 years old, parity as 0, 1–2 and ≥3, and age at first childbirth as <25, 25–29 and ≥30 years old. Education level was categorized as high school or less, two-year college, and university or higher.

In analysis of SNPs, accordance with the Hardy-Weinberg equilibrium was checked in controls using a chi-squared test. The associations between genotype and the risk of breast cancer were estimated by computing OR and the 95% CI from logistic regression analyses. Per allele OR was calculated using 0, 1 or 2 copies of the risk allele (a) as a continuous variable. The reported OR and 95% CI denote the risk difference when increasing the number of risk alleles by one. Two models of analyses were performed, with the first model adjusted only for age and the second model adjusted for factors that were significantly associated with breast cancer risk in this study (multivariate adjustment).

For SNPs associated with breast cancer, we classified subjects as risk allele carriers or non-risk allele carriers and examined associations of lifestyle factors with breast cancer risk in these subgroups. Two models were also used in this analysis, with the second model adjusted for factors that were significantly associated with breast cancer risk in the first model.

All statistical analyses were performed with Statistical Analysis System software JMP version 9.0.3 (SAS Institute).

## Results

A total of 515 patients and 527 controls agreed to participate in the study and gave written informed consent. Of these women, 476 patients (92.4%) and 464 controls (88.8%) returned self-administered questionnaires. In 2 cases, blood samples could not be obtained because of brittle vessels and in another 2 cases SNP genotyping could not be performed because of poor DNA amplification. Thus, the final data set for analysis included 472 patients and 464 controls with completed questionnaires and SNP genotyping.

Adjusted OR with 95% CIs for lifestyle factors are shown in Table 1. BMI ≥24 (vs. 20–21.9) and current or former smoker (vs. never) were associated with a significantly increased risk for breast cancer. Meat intake ≥2 times/week (vs. ≤once/week), mushroom intake (vs. ≤once/week), yellow and green vegetable intake (vs. ≤once/week), coffee intake 2–3 cups/day (vs. <1 cup/day), green tea intake 2–3 cups/day (vs. <1 cup/day), current leisure-time exercise (vs. none), intensity of physical activity in leisure-time exercise 6–23.9 METS/week (vs. 0 METS/week), and university education (vs. high school or less) were all associated with a significantly decreased risk for breast cancer. Height, alcohol intake, age at first menstruation, parity, age at first birth, and familial history of breast cancer have generally been considered to be associated with breast cancer risk, but did not show a significant association in this study.

**Table 1:** Adjusted odds ratios and 95% confidence intervals for lifestyle factors in 472 cases and 464 controls (recruitment period: December 2010 to November 2011)

**Variables**	**Case (n = 472)**		**Control (n = 464)**		**OR**^**a **^**(95% CIs)**	
	**n (%)**		**n (%)**			
Age (year) (mean ± SD)	54.72 ± 12.45		53.56 ± 11.00			
Menopausal status						
Pre	280	(59)	271	(58)		
Post	192	(41)	193	(42)		
Height (cm)						
≤150	95	(20)	78	(17)	1.16	(0.78-1.71)
151-155	147	(32)	145	(32)	Ref.	
156-160	152	(33)	156	(34)	0.99	(0.72-1.36)
>160	72	(15)	81	(18)	0.93	**(**0.63-1.38**)**
Weight (Kg)						
≤50	159	(34)	173	(37)	0.97	(0.69-1.36)
51-55	112	(24)	118	(26)	Ref.	
56 -60	92	(20)	78	(17)	1.24	(0.83-1.85)
>60	104	(22)	93	(20)	1.18	**(**0.80-1.73**)**
BMI (Kg/m^2^)						
20	102	(22)	96	(21)	1.39	(0.96-2.01)
20-21.9	118	(25)	150	(33)	Ref.	
22-23.9	104	(22)	102	(22)	1.28	(0.89-1.84)
≥24	139	(30)	112	(24)	**1.54**	**(1.08-2.19)**
Smoking status						
Never	406	(87)	432	(94)	Ref.	
Current or former	60	(13)	28	(6)	**2.49**	**(1.56-4.06)**
Alcohol drinking						
Never	240	(51)	218	(47)	ref.	
Current or former	231	(49)	243	(53)	0.91	(0.70-1.18)
Alcohol intake (g/day)						
0	240	(51)	218	(48)	ref.	
<5	140	(30)	130	(29)	1.02	(0.75-1.39)
5-10	53	(11)	62	(14)	0.82	(0.54-1.24)
10>	36	(8)	45	(10)	0.75	(0.46-1.21)
Meat intake (times/week)						
≤1	101	(22)	66	(14)	Ref.	
2-4	297	(64)	307	(67)	**0.65**	**(0.45-0.92)**
≥5	67	(14)	88	(19)	**0.51**	**(0.32-0.80)**
Soy intake (times/week)						
≤1	45	(10)	49	(11)	Ref.	
2-4	236	(50)	227	(50)	1.12	(0.72-1.76)
≥5	188	(40)	182	(40)	1.09	(0.69-1.72)
Fish intake (times/week)						
≤1	103	(22)	94	(20)	Ref.	
2-4	297	(64)	314	(68)	0.85	(0.62-1.18)
≥5	67	(14)	53	(11)	1.09	(0.68-1.74)
Eggs intake (times/week)						
≤1	108	(23)	95	(21)	Ref.	
2-4	238	(51)	247	(54)	0.86	(0.62-1.20)
≥5	120	(26)	112	(25)	0.96	(0.66-1.41)
Milk intake (times/week)						
≤1	84	(18)	82	(18)	Ref.	
2-4	157	(34)	135	(30)	1.14	(0.78-1.67)
≥5	226	(48)	238	(52)	0.92	(0.64-1.31)
Fruit intake (times/week)						
≤1	112	(24)	112	(24)	Ref.	
2-4	172	(37)	149	(32)	1.11	(0.79-1.57)
≥5	184	(39)	199	(43)	0.86	(0.61-1.21)
Mushrooms intake (times/week)						
≤1	156	(34)	120	(26)	Ref.	
2-4	247	(53)	261	(57)	**0.73**	**(0.54-0.98)**
≥5	61	(13)	77	(17)	**0.60**	**(0.40-0.91)**
Green and yellow vegetables intake (times/week)						
≤1	47	(10)	28	(6)	Ref.	
2-4	231	(50)	204	(46)	0.66	(0.39-1.09)
≥5	183	(40)	212	(48)	0.48	(0.29-0.80)
Coffee intake (times/week)						
<1	132	(28)	103	(22)	Ref.	
1	154	(33)	158	(34)	0.77	(0.55-1.09)
2-3	135	(29	160	(35)	**0.68**	**(0.48-0.96)**
≥4	45	(10)	40	(9)	0.91	(0.55-1.51)
Green tea intake (times/week)						
<1	200	(43)	182	(40)	Ref.	
1	151	(33)	133	(29)	0.97	(0.71-1.33)
2-3	63	(14)	87	(19)	**0.63**	**(0.43-0.93)**
≥4	48	(10)	55	(12)	0.72	(0.46-1.12)
Leisure-time exercise						
None	254	(54)	214	(46)	Ref.	
Current	214	(46)	248	(54)	**0.70**	**(0.54-0.91)**
Intensity of physical activity^b^ (METs/week)						
0	254	(56)	214	(47)	Ref.	
>6.0	51	(11)	42	(9)	1.05	(0.67-1.65)
6.0-11.9	44	(10)	60	(13)	**0.61**	**(0.39-0.93)**
12.0-23.9	48	(11)	80	(17)	**0.51**	**(0.34-0.75)**
≥24.0	52	(12)	61	(13)	0.70	(0.46-1.07)
Age at menarche (year)						
≤12	140	(30)	201	(44)	0.88	(0.616-1.25)
13	109	(23)	113	(25)	Ref.	
≤14	217	(47)	144	(31)	1.25	(0.882-1.78)
Parity						
0	86	(20)	75	(17)	Ref.	
1-2	247	(57)	265	(59)	0.74	(0.511‒1.06)
≥3	102	(23)	107	(24)	0.76	(0.495‒1.15)
Age at first childbirth (year)						
<25	151	(40)	142	(37)	1.22	(0.89-1.68)
25-29	162	(43)	187	(49)	Ref.	
≥30	63	(17)	50	(13)	1.46	(0.96-2.25)
Breastfeeding						
No	125	(27)	104	(23)	Ref.	
Yes	339	(73)	355	(77)	0.77	(0.57-1.04)
History of benign breast disease						
No	351	(79)	354	(79)	Ref.	
Yes	93	(21)	92	(21)	1.03	(0.74-1.42)
Family history of breast cancer						
No	391	(88)	373	(88)	Ref.	
Yes	53	(12)	52	(12)	0.98	(0.65-1.47)
History of HRT use						
No	424	(92)	412	(90)	Ref.	
Yes	35	(8)	45	(10)	0.76	(0.47-1.21)
Education						
High school or less	259	(55)	196	(43)	Ref.	
Two-year college	144	(31)	144	(31)	0.78	(0.57-1.05)
University	64	(14)	120	(26)	**0.41**	**(0.29-0.59)**

^a^OR is adjusted for age. ^b^Intensity of physical activity in leisure-time exercise. Significant dates are showed in boldface. OR, odds ratio; CI, confidence interval; BMI, body mass index; HRT, hormone replacement therapy.

In analysis of SNPs, deviation from the Hardy-Weinberg equilibrium (P <0.05 by chi square test) was found for rs1800054 and rs1045485, and thus these SNPs were excluded from analysis. The minor allele frequencies were <0.05 for rs4666451 and rs104548, and these SNPs were also excluded, leaving 12 SNPs for analysis. Multivariate ORs were adjusted for factors that were found to be significantly associated with breast cancer: BMI, smoking status, meat intake, mushroom intake, yellow and green vegetable intake, coffee intake, green tea intake, leisure-time exercise and education level.

Age adjusted ORs and multivariate ORs with 95% CIs for independent SNPs in all subjects and in subjects stratified by menopausal status are shown in Table 2. In all women, three SNPs were significantly associated with breast cancer risk in multivariate adjustment: rs2046210 (per allele OR = 1.37 [95% CI:1.11-1.70]), rs3757318 (per allele OR = 1.33 [1.05-1.69] and rs3803662 (per allele = 1.28 [1.07-1.55]). rs2046210 and rs3757318, both of which are located on 6q25.1, are not in strong linkage disequilibrium (LD) (D = 0.68, r2 = 0.21) according to Hap-Map JTP [19]. Among pre-menopausal women, s3803662 (per allele OR = 1.58 [95% CI: 1.17-2.16]) and rs2046210 (per allele OR = 1.70 [95% CI: 1.24-2.35]) were significantly associated with breast cancer risk in multivariate adjustment. Among post-menopausal women, there were no SNPs significantly associated with breast cancer risk.

**Table 2:** Odds ratio with 95% confidence intervals for individual SNPs in all subjects and in subjects stratified by menopausal status

		**All women (n = 936)**					**Premenopausal (n = 385)**					**Postmenopausal (n = 551)**				
**SNP**		**No. of**	**Adjusted OR**^**b**^		**Multivariate OR**^**c**^		**No. of**	**Adjusted OR**^**b**^		**Multivariate OR**^**c**^		**No. of**	**Adjusted OR**^**b**^		**Multivariate OR**^**c**^	
**Gene/location**	**Genotype**^**a**^	**Case/Control**	**OR (95% CI)**		**OR (95% CI)**		**Case/Control**	**OR (95% CI)**		**OR (95% CI)**		**Case/Control**	**OR (95% CI)**		**OR (95% CI)**	
rs1562430	CC	7/4	Ref.		Ref.		2/3	Ref.		Ref.			Ref.		Ref.	
/8q24	TC	96/102	0.54	(0.14-1.85)	0.62	(0.15‒2.32)	33/42	1.24	(0.19-9.85)	1.10	(0.15-10.05)	5/1	0.24	(0.01-1.54)	0.35	(0.02-2.80)
	TT	369/351	0.61	(0.16-2.05)	0.67	(0.16‒2.45)	155/146	1.64	(0.27-12.63)	1.72	(0.24-15.14)	63/60	0.24	(0.01-1.52)	0.29	(0.01-2.25)
	Per allele		1.05	(0.79-1.39)	1.02	(0.75‒1.39)		1.08	(0.81-1.45)	1.62	(1.08-2.44)	214/205	1.07	(0.85-1.36)	0.80	(0.56-1.14)
rs889132	AA	76/91	Ref.		Ref.		34/36	Ref.		Ref.			Ref.		Ref.	
MAP3K1/5q	CA	227/211	1.27	(0.89-1.83)	1.27	(0.86‒1.88)	91/95	0.96	(0.55-1.65)	0.82	(0.45-1.50)	42/55	1.59	(0.98-2.58)	1.57	(0.91-2.76)
	CC	164/160	1.21	(0.83-1.76)	1.21	(0.81‒1.81)	64/61	1.07	(0.60-1.92)	0.98	(0.52-1.84)	136/116	1.35	(0.82-2.23)	1.30	(0.74-2.30)
	Per allele		1.07	(0.89-1.29)	1.07	(0.88‒1.31)		1.08	(0.81-1.45)	1.11	(0.83-1.49)	100/99	1.07	(0.85-1.36)	1.05	(0.81-1.36)
rs13283615	AA	75/75	Ref.		Ref.		29/31	Ref.		Ref.			ref.		ref.	
/8q24	GA	211/206	1.04	(0.71-1.51)	1.09	(0.73‒1.65)	73/80	0.97	(0.53-1.76)	1.13	(0.60-2.17)	46/44	1.10	(0.68-1.79)	1.17	(0.67-2.05)
	GG	180/177	1.03	(0.70-1.51)	1.02	(0.67‒1.55)	86/78	1.14	(0.63-2.05)	1.18	(0.62-2.24)	138/126	0.97	(0.58-1.61)	1.09	(0.61-1.97)
	Per allele		1.01	(0.84-1.21)	1.00	(0.81‒1.22)		1.11	(0.84-1.47)	1.03	(1.00-1.05)	94/99	0.95	(0.74-1.21)	0.99	(0.76-1.28)
rs981782	TT	166/149	Ref.		Ref.		67/64	Ref.		Ref.			Ref.		Ref.	
HCN1/5p12	TG	220/234	0.85	(0.64-1.14)	0.82	(0.60‒1.13)	88/98	0.85	(0.54-1.33)	0.78	(0.48-1.26)	99/85	0.87	(0.59-1.27)	0.83	(0.54-1.29)
	GG	82/76	0.96	(0.66-1.41)	0.88	(0.58‒1.34)	31/28	1.03	(0.56-1.91)	0.97	(0.50-1.90)	132/136	0.93	(0.57-1.52)	0.76	(0.43-1.34)
	Per allele		0.95	(0.79-1.14)	0.97	(0.80‒1.17)		1.00	(0.75-1.35)	1.01	(0.74-1.38)	51/48	0.93	(0.73-1.18)	0.86	(0.66-1.13)
rs3803662	CC	74/91	Ref.		Ref.		24/42	Ref.		Ref.			Ref.		Ref.	
TNRC9/16q12	TC	230/227	1.25	(0.88-1.79)	1.32	(0.89‒1.96)	89/96	1.59	(0.90-2.85)	1.50	(0.81-2.80)	50/49	1.08	(0.68-1.72)	1.25	(0.73-2.16)
	TT	160/142	1.41	(0.97-2.08)	**1.61**	**(1.06‒2.45)**	72/53	**2.29**	**(1.25-4.26)**	**2.29**	**(1.20-4.46)**	141/131	1.04	(0.63-1.71)	1.27	(0.72**-**2.24)
	Per allele		1.18	(0.98-1.42)	**1.28**	**(1.07‒1.55)**		**1.54**	**(1.15-2.09)**	**1.58**	**(1.17-2.16)**	88/89	1.00	(0.78-1.28)	1.07	(0.83**-**1.39)
rs381798	TT	339/347	Ref.		Ref.		138/140	Ref.		Ref.			Ref.		Ref.	
LSP1/11p15.5	CT	120/107	1.14	(0.85-1.55)	1.07	(0.77‒1.49)	46/49	0.92	(0.58-1.48)	1.00	(0.60-1.68)	201/207	1.30	(0.87-1.94)	1.18	(0.75-1.86)
	CC	10/5	2.04	(0.72-6.60)	1.63	(0.52‒5.66)	4/1	3.98	(0.58-78.39)	3.29	(0.42-68.89)	74/58	1.65	(0.46-6.55)	1.39	(0.32-6.31)
	Per allele		1.19	(0.91-1.56)	1.11	(0.83‒1.49)		1.07	(0.70-1.64)	1.21	(0.77-1.90)	6/4	1.27	(0.90-1.81)	1.14	(0.78-1.66)
rs2046210	GG	213/244	Ref.		Ref.		83/107	Ref.		Ref.			Ref.		Ref.	
ESR1/6q25.1	AG	194/185	1.21	(0.92-1.59)	1.22	(0.90‒1.64)	78/72	1.41	(0.92-2.17)	1.63	(1.03-2.61)	130/137	1.11	(0.78-1.59)	0.99	(0.67-1.48)
	AA	61/34	**2.03**	**(1.29-3.25)**	**2.16**	**(1.32‒3.59)**	27/14	**2.46**	**(1.23-5.10)**	**2.93**	**(1.40-6.40)**	116/113	1.69	(0.93-3.14)	1.69	**(**0.84**-**3.50**)**
	Per allele		**1.34**	**(1.10-1.63)**	**1.37**	**(1.11‒1.70)**		**1.49**	**(1.10-2.03)**	**1.70**	**(1.24-2.35)**	34/20	1.23	(0.95-1.59)	1.14	**(**0.86**-**1.51**)**
rs909116	CC	166/178	Ref.		Ref.		71/64	Ref.		Ref.			Ref.		Ref.	
LSP/11p15.5	CT	225/228	1.08	(0.81-1.43)	1.04	(0.77‒1.42)	88/106	0.76	(0.49-1.18)	0.90	(0.55-1.47)	95/114	1.36	(0.94-1.97)	1.20	(0.79-1.83)
	TT	79/57	1.49	(0.99-2.24)	1.40	(0.90‒2.19)	30/23	1.21	(0.64-2.30)	1.23	(0.62-2.48)	137/122	**1.72**	**(1.02-2.90)**	1.69	(0.94-3.09)
	Per allele		1.18	(0.97-1.42)	1.15	(0.93‒1.41)		0.98	(0.72-1.32)	1.11	(0.81-1.52)	49/34	**1.32**	**(1.03-1.69)**	1.24	(0.95-1.63)
rs30099	CC	225/216	Ref.		Ref.		93/84	Ref.		Ref.			Ref.		Ref.	
/5q	TC	205/198	0.82	(0.52-1.29)	1.08	(0.80‒1.45)	82/84	0.87	(0.57-1.33)	0.96	(0.61-1.53)	132/132	1.08	(0.76-1.54)	1.21	(0.80-1.83)
	TT	42/50	0.99	(0.76-1.30)	0.86	(0.52‒1.41)	15/25	0.53	(0.26-1.06)	0.51	(0.24-1.08)	123/114	1.12	(0.61-2.06)	1.19	(0.58-2.45)
	Per allele		0.93	(0.76-1.13)	0.98	(0.79‒1.22)		0.78	(0.57-1.06)	0.85	(0.92-1.16)	27/25	1.04	(0.81-1.36)	1.12	(0.83-1.50)
rs2981282	CC	220/226	Ref.		Ref.		86/94	Ref.		Ref.			Ref.		Ref.	
FGFR2 /10q26	TC	210/190	1.15	(0.87-1.50)	1.19	(0.89‒1.60)	91/81	1.23	(0.81-1.87)	1.48	(0.94-2.35)	134/132	1.10	(0.77-1.58)	1.08	(0.72-1.62)
	TT	41/45	0.92	(0.58-1.47)	0.84	(0.50‒1.40)	13/17	0.89	(0.41-1.92)	1.07	(0.46-2.50)	119/109	0.95	(0.53-1.71)	0.76	(0.38-1.48)
	Per allele		1.03	(0.84-1.25)	1.02	(0.82‒1.27)		1.04	(0.75-1.43)	1.27	(0.91-1.78)	28/28	1.04	(0.80-1.34)	0.94	(0.71-1.24)
rs795399	TT	255/249	Ref.		Ref.		90/107	Ref.		Ref.			Ref.		Ref.	
IGF1/12q23.2	CT	180/173	0.84	(0.51-1.36)	1.05	(0.78‒1.41)	82/65	1.49	(0.97-2.30)	1.56	(0.98-2.48)	165/142	0.80	(0.56-1.15)	0.78	(0.52-1.18)
	CC	34/41	1.03	(0.78-1.35)	0.85	(0.49‒1.45)	15/20	0.86	(0.41-1.77)	1.04	(0.46-2.27)	98/108	0.87	(0.44-1.70)	0.93	(0.43-1.99)
	Per allele		0.96	(0.79-1.18)	0.97	(0.78‒1.21)		1.13	(0.83-1.55)	1.25	(0.91-1.72)	19/21	0.87	(0.66-1.14)	0.88	(0.66-1.17)
rs3757318	GG	249/281	Ref.		Ref.		95/111	Ref.		Ref.			Ref.		Ref.	
ESR1/6q25.1	AG	182/162	1.27	(0.97-1.67)	**1.25**	**(0.93‒1.69)**	76/72	1.25	(0.82-1.91)	1.22	(0.77-1.92)	154/170	1.27	(0.88-1.81)	1.20	(0.79-1.80)
	AA	34/19	**2.01**	**(1.13-3.68)**	**2.05**	**(1.09‒3.97)**	14/8	2.02	(0.83-5.25)	1.90	**(**0.73**-**5.25**)**	106/90	1.96	(0.92-4.37)	2.14	**(**0.88-5.49**)**
	Per allele		**1.34**	**(1.08-1.66)**	**1.33**	**(1.05‒1.69)**		1.30	(0.93-1.83)	1.34	**(**0.95**-**1.91**)**	20/11	1.32	(1.00-1.76)	1.27	**(**0.93-1.75**)**

^a^Alleles on upper line are common alleles; ^b^Adjusted for age; ^c^Multivariate adjusted for age, BMI, smoking, meat intake, mushroom intake, green and yellow vegetable intake, coffee intake, green tea intake, leisure-time exercise and education. Significant dates are showed in boldface. OR, odds ratio; CI, confidence interval.

A subgroup analysis was performed for rs2046210 and rs3757318. For rs2046210, leisure time exercise was associated with a significantly decreased breast cancer risk in risk allele carriers (AA + AG), but not in non-risk allele carriers (GG). In contrast, BMI ≥ 24 and current smoking were associated with a significantly increased breast cancer in non-risk allele carriers (GG), but not in risk allele carriers (AA + AG). Intensity of physical activity in leisure exercise of 12.0-23.9 METS/week and university education were associated with breast cancer risk in risk allele and non-risk allele carriers (Table 3). For rs3757318, BMI ≥ 24 was associated with a significantly increased breast cancer risk in risk allele carriers (GG), but not in risk allele carriers (AA + AG). University education and current smoking were associated with breast cancer risk in risk allele and non-risk allele carriers (Table 4).

**Table 3:** Age-adjusted odds ratio and multivariate adjusted odds ratio with 95% confidence intervals for lifestyle factors in rs2046210

		**Risk allele carriers (AA + AG) n = 474**							**Non-risk allele carriers (GG) n = 457**						
		**Case n = 255/Control n = 219**							**Case n = 213/Control n = 244**						
		**n/n**	**OR**^**a **^**(95% CI)**		**p**	**OR**^**b **^**(95% CI)**		**p**	**n/n**	**OR**^**a **^**(95% CI)**		**p**	**OR**^**c **^**(95% CI)**		**p**
Age (years)		54.0/53.9							55.8/53.2						
Menopausal status	Pre	148/133							130/137						
	Post	107/86							83/107						
Height (cm)	≤150	40/39	1.03	(0.58-1.83)	0.93	0.96	(0.53-1.74)	0.89	55/39	1.34	(0.78-2.9)	0.29	1.19	(0.66-2.14)	0.57
	151-155	76/77	Ref.			Ref.			68/68	Ref.			Ref.		
	156-160	89/66	1.38	(0.88-2.16)	0.16	1.44	(0.91-2.29)	0.12	63/89	0.76	(0.48-1.3)	0.27	0.89	(0.53-1.48)	0.64
	>160	46/34	1.41	(0.81-2.47)	0.23	1.62	(0.91-2.91)	0.10	25/47	0.59	(0.32-1.08)	0.09	0.51	**(**0.25-0.99**)**	0.05
BMI (Kg/m^2^)	20	59/46	1.27	(0.75-2.14)	0.37	1.13	(0.67-1.94)	0.64	43/50	1.62	(0.93-2.81)	0.09	1.54	(0.84-2.82)	0.16
	20-21.9	69/67	Ref.			Ref.			48/82	Ref.			Ref.		
	22-23.9	58/50	1.09	(0.66-1.80)	0.75	0.97	(0.58-1.63)	0.92	43/52	1.40	(0.82-2.40)	0.22	1.47	(0.83-2.63)	0.19
	≥24	65/53	1.17	(0.71-1.94)	0.53	1.09	(0.65-1.82)	0.74	74/59	**2.07**	**(1.26-3.43)**	**<0.01**	**1.91**	**(1.11-3.29)**	**0.02**
Smoking status	Never	222/201	Ref.			Ref.			180/230	Ref.			Ref.		
	Current or former	29/15	1.78	(0.93-3.51)	0.08	1.61	(0.83-3.21)	0.16	31/13	**3.82**	**(1.94-7.98)**	**<0.01**	**3.86**	**(1.87-8.37)**	**<0.01**
Alcohol drinking	Never	129/107	Ref.			Ref.			108/111	Ref.			Ref.		
	Current or former	125/109	0.97	(0.67-1.40)	0.97	1.07	(0.73-1.57)	0.74	105/133	0.91	(0.62-1.33)	0.61	0.87	(0.56-1.33)	0.51
Alcohol intake (g/day)	0	129/107	Ref.			Ref.			108/111	Ref.			Ref.		
	<5	75/56	1.12	(0.72-1.74)	0.61	1.22	(0.78-1.92)	0.39	64/73	0.99	(0.64-1.54)	0.98	0.98	(0.60-1.61)	0.94
	5-10	28/32	0.75	(0.42-1.34)	0.34	0.88	(0.49-1.60)	0.68	25/30	0.94	(0.51-1.72)	0.85	0.92	(0.46-1.80)	0.80
	10>	20/19	0.88	(0.44-1.74)	0.71	0.94	(0.46-1.89)	0.85	16/26	0.70	(0.35-1.38)	0.31	0.55	(0.24-1.22)	0.14
Leisure-time exercise	No	143/97	Ref.			Ref.			110/116	Ref.			Ref.		
	Yes	110/121	**0.62**	**(0.43-0.89)**	**0.01**	**0.60**	**(0.41-0.87)**	**<0.01**	101/127	0.77	(0.52-1.12)	0.17	0.74	(0.49-1.11)	0.14
Intensity of physical activity^d^ (met/week)	0	143/99	Ref.			Ref.			109/119	Ref.			Ref.		
	>6.0	25/23	0.79	(0.42-1.48)	0.45	0.72	(0.38-1.37)	0.32	25/19	1.35	(0.70-2.63)	0.37	1.20	(0.59-2.48)	0.61
	6.0-11.9	20/28	**0.49**	**(0.26-0.92)**	**0.03**	**0.46**	**(0.24-0.86)**	**0.02**	22/32	0.63	(0.34-1.17)	0.15	0.66	(0.34-1.28)	0.22
	12.0-23.9	27/36	**0.52**	**(0.29-0.91)**	**0.02**	**0.53**	**(0.30-0.94)**	**0.03**	21/44	**0.48**	**(0.26-0.85)**	**0.01**	**0.45**	**(0.24-0.83)**	**0.01**
	≥24.0	30/32	0.65	(0.37-1.14)	0.13	0.68	(0.38-1.20)	0.18	22/29	0.74	(0.40-1.38)	0.35	0.70	(0.36-1.36)	0.30
Age at menarche	≤12	70/92	0.73	(0.45-1.19)	0.73	0.72	(0.44-1.19)	0.20	68/109	1.07	(0.63-1.81)	0.80	0.98	(0.56-1.70)	0.93
(year)	13	66/55	Ref.			Ref.			43/58	Ref.			Ref.		
	≤14	116/68	1.20	(0.74-1.93)	1.20	1.15	(0.71-1.89)	0.57	99/75	1.32	(0.78-2.25)	0.29	1.62	(0.93-2.84)	0.09
Parity	0	54/35	Ref.			Ref.			31/40	Ref.			Ref.		
	1-2	123/122	0.63	(0.38-1.04)	0.07	0.66	(0.40-1.10)	0.11	124/143	0.95	(0.55-1.64)	0.85	1.12	(0.61-2.09)	0.71
	≥3	54/53	0.65	(0.36-1.15)	0.14	0.65	(0.36-1.17)	0.15	46/53	0.94	(0.50-1.76)	0.84	1.29	(0.64-2.62)	0.48
Age at first childbirth	<25	78/68	1.21	(0.77-1.90)	0.40	1.08	(0.68-1.71)	0.74	72/74	1.22	(0.78-1.91)	0.38	1.17	(0.71-1.91)	0.54
(year)	25-29	87/89	Ref.			Ref.			75/97	Ref.			Ref.		
	≥30	33/22	1.55	(0.84-2.90)	0.16	1.45	(0.77-2.76)	0.25	30/28	1.39	(0.77-2.54)	0.27	1.77	(0.92-3.45)	0.09
Breastfeeding	No	72/51	Ref.			Ref.			51/53	Ref.			Ref.		
	Yes	178/165	0.76	(0.50-1.16)	0.21	0.77	(0.50-1.17)	0.22	159/189	0.83	(0.53-1.30)	0.42	1.02	(0.62-1.69)	0.93
Family history of	No	209/180	Ref.			Ref.			178/192	Ref.			Ref.		
Breast cancer	Yes	31/24	1.11	(0.63-1.97)	0.55	1.12	(0.63-2.00)	0.71	22/28	0.82	(0.45-1.50)	0.75	1.07	(0.57-2.05)	0.83
Education	High school or less	135/99	Ref.			Ref.			123/96	Ref.			Ref.		
	Two-year college	81/63	0.93	(0.61-1.42)	0.74	0.95	(0.62-1.47)	0.83	60/81	**0.62**	**(0.40-0.95)**	**0.03**	**0.59**	**(0.37-0.94)**	**0.03**
	University	36/55	**0.48**	**(0.29-0.79)**	**<0.01**	**0.48**	**(0.29-0.79)**	**<0.01**	28/65	**0.35**	**(0.21-0.59)**	**<0.01**	**0.38**	**(0.22-0.66)**	**<0.01**

^a^OR is adjusted for age.

^b^Multivariate adjusted for leisure-time exercise and education. ^c^Multivariate adjusted for BMI, smoking state, intensity of physical activity and education.

^d^Intensity of physical activity and education. Significant dates are showed in boldface.

OR, odds ratio; CI, confidence interval; BMI, body mass index.

**Table 4:** Age-adjusted odds ratio and multivariate adjusted odds ratio with 95% confidence intervals for lifestyle factors in rs3757318

		**Risk allele carriers(AA + AG) n = 397**							**non-risk allele carriers(GG) n = 530**						
		**Case n = 216/Control n = 181**							**Case n = 249/Control n = 281**						
		**n/n**	**OR**^**a **^**(95%CI)**		**p**	**OR**^**b **^**(95% CI)**		**p**	**n/n**	**OR**^**a **^**(95% CI)**		**p**	**OR**^**c **^**(95% CI)**		**p**
Age (years)		54.23/53.30							55.28/53.76						
Menopausal status	Pre	124/101							154/170						
	Post	92/80							95/111						
Height (cm)	≤150	36/28	1.24	(0.66-2.34)	0.50	1.46	(0.68-3.16)	0.33	58/50	1.07	(0.65-1.77)	0.78	1.01	(0.60-1.69)	0.98
	151-155	62/63	Ref.			Ref.			84/80	Ref.			ref.		
	156-160	78/51	1.57	(0.96-260)	0.07	1.57	(0.86-2.90)	0.14	72/105	0.68	(0.44-1.05)	0.08	0.73	(0.47-1.15)	0.18
	>160	36/38	1.00	(0.55-1.80)	0.99	0.58	(0.26-1.24)	0.16	34/43	0.80	(0.46-1.39)	0.43	0.89	(0.50-1.59)	0.70
BMI(Kg/m^2^)	<20	48/37	1.36	(0.77-2.40)	0.26	1.11	(0.54-2.29)	0.77	54/59	1.57	(0.95-2.59)	0.06	1.60	(0.95-2.69)	0.08
	20-21.9	59/60	Ref.			Ref.			54/90	Ref.			Ref.		
	22-23.9	47/35	1.35	(0.77-2.40)	0.24	1.57	(0.80-3.12)	0.19	57/66	1.41	(0.86-2.30)	0.40	1.29	(0.78-2.14)	0.32
	≥24	57/48	1.18	(0.69-2.01)	0.51	1.14	(0.60-2.17)	0.68	81/63	**2.08**	**(1.29-3.37)**	**<0.01**	**1.89**	**(1.16-3.10)**	**0.01**
Smoking status	Never	186/168	Ref.			Ref.			214/262	Ref.			Ref.		
	Current or former	25/11	**2.15**	**(1.05-4.71)**	**0.04**	**2.73**	**(1.07-7.65)**	**0.04**	34/17	**2.82**	**(1.53-5.40)**	**<0.01**	**2.39**	**(1.27-4.65)**	**<0.01**
Alcohol drinking	Never	114/90	Ref.			Ref.			124/127	Ref.			Ref.		
	Current or former	101/89	0.93	(0.62-1.39)	0.71	0.99	(0.60-1.65)	0.97	125/153	0.90	(0.63-1.28)	0.55	0.95	(0.65-1.38)	0.78
Alcohol intake	0	114/90	Ref.			Ref.			124/127	Ref.			Ref.		
(g/day)	<5	59/45	1.08	(0.67-1.76)	0.75	1.12	(0.61-2.04)	0.72	78/84	1.01	(0.67-1.51)	0.98	1.11	(0.72-1.70)	0.64
	5-10	27/27	0.81	(0.44-1.49)	0.50	0.88	(0.41-1.90)	0.75	25/35	0.79	(0.44-1.41)	0.43	0.89	(0.49-1.63)	0.71
	10>	13/16	0.65	(0.29-1.41)	0.27	0.78	(0.27-2.14)	0.63	22/29	0.82	(0.44-1.52)	0.54	0.66	(0.33-1.28)	0.22
Leisure-time	No	122/80	Ref.			Ref.			127/133	Ref.			Ref.		
Exercise	Yes	93/101	**0.58**	**(0.39-0.87)**	**<0.01**	0.78	(0.47-1.27)	0.32	119/146	0.82	(0.58-1.17)	0.27	0.84	(0.59-1.21)	0.35
Intensity of physical activity^d^(met/week)	0	122/81	Ref.			Ref.			126/137	Ref.			Ref.		
	>6.0	23/17	0.87	(0.44-1.76)	0.70	1.62	(0.68-4.03)	0.28	28/25	1.24	(0.68-2.27)	0.48	1.19	(0.64-2.25)	0.58
	6.0-11.9	21/25	0.55	(0.28-1.04)	0.07	0.58	(0.27-1.21)	0.15	23/34	0.68	(0.37-1.22)	0.20	0.69	(0.37-1.28)	0.24
	12.0-23.9	19/32	**0.39**	**(0.20-0.73)**	**<0.01**	0.73	(0.33-1.56)	0.41	29/48	0.63	(0.37-1.06)	0.08	0.62	(0.36-1.06)	0.08
	≥24.0	23/26	0.56	(0.29-1.06)	0.07	0.67	(0.31-1.42)	0.29	27/35	0.79	(0.45-1.39)	0.42	0.84	(0.47-1.50)	0.55
Age at menarche	≤12	63/73	1.00	(0.59-1.70)	0.99	1.67	(0.85-3.33)	0.14	73/127	0.77	(0.47-1.24)	0.28	0.74	(0.45-1.22)	0.24
(year)	13	52/51	Ref.			Ref.			57/61	Ref.			Ref.		
	≤14	99/56	1.39	(0.82-2.35)	0.22	1.74	(0.90-3.37)	0.10	115/88	1.12	(0.70-1.81)	0.63	1.02	(0.62-1.68)	0.92
Parity	0	49/24	Ref.			Ref.			37/50	Ref.			Ref.		
	1-2	110/105	**0.48**	**(0.27-0.84)**	**<0.01**	0.55	(0.19-1.54)	0.25	132/160	0.98	(0.60-1.62)	0.95	1.19	(0.70-2.05)	0.52
	≥3	36/48	**0.34**	**(0.17-0.65)**	**<0.01**	0.35	(0.12-1.04)	0.06	65/58	1.36	(0.77-2.40)	0.29	1.74	(0.95-3.21)	0.07
Age at first childbirth	<25	60/60	1.05	(0.64-1.71)	0.86	0.97	(0.56-1.66)	0.90	88/82	1.35	(0.89-2.05)	0.15	1.19	(0.77-1.84)	0.43
(year)	25-29	72/77							88/110	Ref.			Ref.		
	≥30	34/19	**1.96**	**(1.03-3.80)**	**0.04**	1.82	(0.88-3.85)	0.11	29/31	1.17	(0.66-2.10)	0.59	1.27	(0.69-2.33)	0.45
Breastfeeding	No	65/38	Ref.			Ref.			59/65	Ref.			Ref.		
	Yes	150/143	**0.60**	**(0.38-0.95)**	**0.03**	0.93	(0.36-2.43)	0.89	183/211	0.91	(0.61-1.38)	0.67	1.07	(0.69-1.65)	0.77
Family history of	No	173/143	Ref.			Ref.			212/229	Ref.			Ref.		
Breast cancer	Yes	24/19	1.04	(0.55-2.00)	0.79	1.30	(0.56-3.07)	0.54	28/33	0.91	(0.53-1.57)	0.93	0.90	(0.51-1.58)	0.72
Education	High school or less	113/80	Ref.			Ref.			144/115	Ref.			Ref.		
	Two-year college	74/54	0.99	(0.62-1.57)	0.96	1.02	(0.58-1.79)	0.94	66/90	**0.60**	**(0.40-0.91)**	**0.01**	**0.63**	**(0.42-0.96)**	**0.03**
	University	27/45	**0.43**	**(0.24-0.76)**	**<0.01**	**0.33**	**(0.16-0.67)**	**0.00**	36/74	**0.40**	**(0.25-0.64)**	**<0.01**	**0.45**	**(0.28-0.73)**	**<0.01**

^a^OR is adjusted for age.

^b^Multivariate adjusted for smoking state, leisure-time exercise, party, age of first children, breastfeeding and education. ^c^Multivariate adjusted for BMI, smoking state, and education. ^d^Intensity of physical activity and education. Significant dates are showed in boldface. OR, odds ratio; CI, confidence interval; BMI, body mass index.

## Discussion

Associations of breast cancer risk with lifestyle factors and SNPs alone and in combination were examined in a case–control study in 472 patients and 464 controls. Reproductive factors such as early age at first menstruation, late age at menopause, late age at first birth, nulliparity, and no breastfeeding have been associated with an increase in breast cancer risk [20], including in the Japanese population [21]. In our study, parity and breastfeeding showed a tendency for an association with decreased breast cancer risk, but this association was not significant; and age at first menstruation, age at first birth, and age at menopause were not significantly associated with breast cancer risk. In most previous studies, comparisons were made using categories for age at first menstruation of 12–13 and >15 years old [22] and age at first birth of ≤24 and >30 years old [23]. In the current study, the sample sizes for women who were >15 years old at first menstruation and >30 years old at first birth were too small to analyze correctly, which is a limitation in the study.

The associations of food and nutrition with breast cancer risk have been summarized by the WCRF/AICR [3]. The effects of some foods on breast cancer are unclear, but we found that intake of meat, mushrooms, yellow and green vegetables, coffee and green tea was associated with decreased breast cancer risk. The evidence that alcohol is associated with breast cancer was judged to be “convincing” by the WCRF/AICR, but we did not find this association, which is consistent with other Japanese studies. The frequency and amount of food consumption depends on cultures and customs in different countries, and this may cause the factors and threshold level for breast cancer risk to also vary in the respective countries.

Cigarette smoking [24,25] is also considered to be associated with increased breast cancer risk, while leisure-time exercise [26] is associated with decreased breast cancer risk, including in the Japanese population. The mean BMI of the Asian population, including the Japanese population, is lower than that in non-Asians [27]. However, we found that BMI ≥24 is associated with increased breast cancer risk, as found in other Japanese studies [28].

A high education level has been associated with increased breast cancer risk, but this may be explained by highly educated women having a high rate of nulliparity and being older at first birth. However, in Japan, social advances and college attendance have only become more common for women in recent years, and thus education level may not correlate well with social status and an unwed state. Instead, more highly educated women are more likely to be involved in preventive health behavior such as exercise, non-smoking, no alcohol intake and avoidance of obesity, compared to women with less education, and some studies have associated a higher education level with a decreased breast cancer risk [29,30].

The current study has several limitations. First, selection bias may have influenced the results because we enrolled women who underwent breast cancer screening as controls. In Japan, the rate of breast cancer screening was no more than about 25% in 2010 [31]. Thus, women who undergo screening may have more interest in trying to maintain their health and may have a family history of cancer, which may have eliminated the significant association of a family history of breast cancer with breast cancer risk in our study. Second, recall bias may have influenced the results because of the use of self-administered questionnaires. In particular, data from patients might lack accuracy because their answers reflected their behavior before diagnosis.

In all subjects, 3 of the 16 SNPs analyzed in the study were significantly associated with breast cancer risk. These included rs2046210 and rs3757318, which are located at 6q25.1, in proximity to the estrogen receptor 1 gene (ESR1). ESR1 encodes an estrogen receptor (ERα), a ligand-activated transcription factor composed of several domains important for hormone binding, DNA binding, and activation of transcription [32]. ERα is mainly expressed in the uterus, ovary, bone, and breast in females [33], ERα is also overexpressed in 60-70% of cases of breast cancer and is involved in the disease pathology. Although these SNPs are located in the same chromosome region, they are not in strong LD based on the HapMap Project. Potential involvement of both SNPs in regulation of ESR1 is unclear [14,34]. rs2046210 is located 29 kb upstream of the first untranslated exon. The risk allele frequency of rs2046210 is 33.3% in Europeans (HapMap-CEU), 37.8% in Chinese (Hap Map-HCB) and 30.0% in Japanese (HapMap-JTP) [19]. Our result indicated a 27% risk allele frequency, which was about the same as that in HapMap-JTP. Thus, the risk allele frequency of Asians differs little from that of Europeans. Several studies have associated rs2046210 with breast cancer risk [15,34-36]. Guo et al. found a significant association between rs2046210 and breast cancer risk in the overall population (per allele OR 1.14, 95% CI =1.10–1.18) and in Asians (per allele OR 1.27, 95% CI =1.23–1.31) and Europeans (per allele OR 1.09, 95% CI =1.07–1.12), indicating that rs2046210 has a larger effect in Asians [34]. Our results also suggest that rs2046210 is significantly associated with breast cancer risk in Japanese subjects.

Turnbull et al. first reported a significant association of rs3757318 with breast cancer risk [11]. rs3757318 is located 200 kb upstream of ESR1. The risk allele frequency of rs3757318 is 6.6% in Europeans (HapMap-CEU), 33% in Chinese (HapMap-HCB) and 25% in Japanese (HapMap-JTP) [19]. We found a 22% risk allele frequency, consistent with HapMap-JTP. Thus, the risk allele frequency for rs3757318 varies between Europeans and Asians. In an analysis of the association between rs2046210 and rs12662670 as a surrogate for rs3757318 and breast cancer risk, Heins et al. found that that per allele OR for rs3757318 was higher in Asians (1.29, 95% CI 1.19–1.41) than in Europeans (1.12, 95% CI 1.08–1.17) [31]. These results suggest that screening for the rs3757318 genotype may be important in Asian women.

We also found that SNPs associated with breast cancer differed with regard to menses state, with rs2046210 and rs3803662 associated with breast cancer risk in premenopausal women. rs3803662 lies 8 kb upstream of TNRC9 and was found to have a significant association with breast cancer risk by Easton et al. [12]. TNRC9 is located on chromosome 16q12 and consists of seven exons. The protein encoded by this gene is a member of the high mobility group box (HMG-box) family. TNRC9 is expressed in brain and breast tissue, and has a higher expression level in breast cancer compared to that in normal tissue [37]. The risk allele frequency of rs3803662 is 24% in Europeans (HapMap-CEU), 72% in Chinese (HapMap-HCB) and 60% in Japanese (HapMap-JTP) [19]. Thus, Asian populations have a higher risk allele frequency than Europeans. However, Chen et al. found that rs3803662 was significantly associated with breast cancer in Europeans [17], but that this relationship was unclear in Asians [38]. Among the breast cancer-associated SNPs found in the current study, rs2046210 and rs3757318 are located near ESR1 and are related to breast cancer risk in Asians. To examine whether lifestyle factors associated with breast cancer risk vary in risk allele and non-risk allele carriers, we performed a subgroup analysis. Leisure-time exercise were associated with a decreased breast cancer risk in rs2046210 risk allele carriers. Although low-penetrance susceptibility SNPs may confer only a small effect on breast cancer risk alone, the risk for development of breast cancer in a risk allele carrier is about 1.2-1.3 fold higher than that in non-carriers. However, our results suggest that risk allele carriers can reduce their breast cancer risk through exercise, whereas obesity and smoking may increase breast cancer risk in non risk-allele carriers. An understanding of the mechanisms underlying the different lifestyle factors associated with breast cancer in rs2046210 and rs3757318 risk allele and non-risk allele carriers may clarify the effects of these SNPs located near ESR1. Examination of interactions between SNPs and lifestyle factors in a larger Japanese population is needed to confirm the current findings for SNPs, lifestyle factors and breast cancer.

## Conclusions

This case–control study showed that rs2046210 and rs3757318 located near the ESR1 gene and rs3808662 located on TNRC9 are associated with breast cancer risk in Japanese women. Our results suggest that leisure-time exercise can reduce the breast cancer risk in rs2046210 risk allele carriers, whereas smoking and obesity may increase the breast cancer risk in non-risk allele carriers. Further studies are required to confirm the validity of the association of these SNPs and lifestyle factors with breast cancer risk in the Japanese population.

## Abbreviations

SNPs: Single nucleotide polymorphisms; WCRF/AICR: World Cancer Research Fund/American Institute for Cancer Research; NIC: National Cancer Institute; GWAS: Genome-wide association studies; LD: Linkage disequilibrium; BMI: Body mass index; MET: Metabolic equivalent; OR: Odds ratio; CI: Confidence interval; ERα: estrogen receptor α.

## Competing interests

The authors declare that they have no competing interests.

## Authors’ contributions

NT designed the study. TM carried out genotyping, performed statistical analysis, and wrote the manuscript with NT. KN participated in genotyping and statistical analysis. TN, TI, TM, TS, JM, HD, SI, HK, KK, YI and YO obtained informed consent from subjects, collected blood samples and data from subjects, and provided advice on the study. YK designed the study and served as an advisor. All authors read and approved the final manuscript.

## Pre-publication history

The pre-publication history for this paper can be accessed here:

http://www.biomedcentral.com/1471-2407/13/565/prepub
